# Dietary Exposure Assessment of Rare Earth Elements in the Chinese Population

**DOI:** 10.3390/ijerph192315583

**Published:** 2022-11-24

**Authors:** Daoyuan Yang, Haixia Sui, Weifeng Mao, Yibaina Wang, Dajin Yang, Lei Zhang, Zhaoping Liu, Ling Yong, Yan Song

**Affiliations:** NHC Key Laboratory of Food Safety Risk Assessment, China National Center for Food Safety Risk Assessment, Beijing 100022, China

**Keywords:** rare earth elements, dietary exposure, Chinese population, cumulative risk assessment, exposure assessment, lanthanum, cerium, yttrium

## Abstract

Rare earth elements (REEs) are widely found in foods. A high intake of REEs may have associations with adverse effects on human health. This study aimed to investigate the concentrations of REEs in foods in China and to assess the risk of dietary REEs exposure in the Chinese population. The mean concentrations of the total REEs in 27,457 food samples from 11 food categories ranged from 0.04 to 1.41 mg/kg. The daily mean dietary exposure of the total REEs was 1.62 μg/kg BW in the general Chinese population and ranged from 1.61 to 2.80 μg/kg BW in different sex–age groups. The high consumer exposure (95th percentile, P95) was 4.83 μg/kg BW, 9.38% of the temporary ADI (tADI) of REEs (51.5 μg/kg BW). None of the P95 exposure exceeded the tADI in all of the sub-groups. Lanthanum, cerium, and yttrium accounted for approximately 63% of the total exposure of the 16 REEs. The hazard index of 16 REEs was far below 1. Therefore, the health risk of dietary REEs exposure in the general Chinese population was low. No cumulative risk was found for the 16 REEs in China. The results indicate there was no need to stipulate the limits of REEs in foods.

## 1. Introduction

Rare earth elements (REEs) are a series of lanthanide elements with atomic numbers 57 through 71 on the periodic table, as well as scandium (Sc) and yttrium (Y), which share similar chemical and physical properties and tend to exist in the same ore deposits [1,2,3,4]. REEs are commonly divided into light REEs (LREEs) and heavy REEs (HREEs). LREEs are elements with smaller atomic masses, higher solubility, and higher alkalinity, including lanthanum (La), cerium (Ce), praseodymium (Pr), neodymium (Nd), promethium (Pm), samarium (Sm), and Sc. HREEs are elements with higher atomic masses, lower solubility, and lower alkalinity, including europium (Eu), gadolinium (Gd), terbium (Tb), dysprosium (Dy), holmium (Ho), erbium (Er), thulium (Tm), ytterbium (Yb), lutecium (Lu), and Y [5,6]. REEs are abundant in the Earth’s crust and the crustal abundance of Ce is comparable to the common metal copper; Ce, La, Nd, and Y have the highest resources and production [7].

Human activities have been the main source of REEs in our environment for years, eventually contaminating the surface of the Earth. The mining, extracting, and processing of REE ores can release REEs into the environment. Meanwhile, because of their superior physical and chemical properties, REEs are widely used in industries, agriculture, and medical technologies [8]. The applications of REEs as fertilizers and feed additives have been extensively reviewed [9]. China has the largest REE reserve across the world (approximately 44 million tons) and was the largest REE producer in 2021 (about 60% of the production); China was also the first country in the world to use REEs as fertilizers in crop production and as performance-enhancers in animal nutrition [10,11]. It was reported that the total amount of REEs applied in agriculture was 54,100 tons in China until 2007 [12]. In 2020, the European Commission regulation (2020/1370) approved the use of lanthanide citrate as a zootechnical additive for weaned piglets [13]. Besides being intentionally added as fertilizers and feed additives, REEs can enter the environment by using phosphate fertilizers produced from phosphate minerals that contain REEs [5]. In Canada, the application of phosphate fertilizers steadily increased from 1995 and was approximately 750,000 tons per year in 2011/2012 [14]. In Brazil, approximately 12,000 tons of Ce were estimated to enter the soil by using phosphate fertilizers in 2014 alone [5].

With the large-scale and sharp increase in REE mining and applications, the contamination levels of REEs have increased in the soil, water, and air, and consequently in the food chain [15,16]. Food ingestion is an important exposure pathway of REEs for humans, which may induce adverse effects on the human body [15]. Relative to other common metals, only a few toxicological studies of REEs are available in the literature [17]. However, the limited toxicological data from animal studies and human investigations demonstrate that REEs could induce tissue-specific bioaccumulation after entering the human body and induce damage to the nervous system, reproductive system, and immune system [18,19,20,21]. Meanwhile, the existing toxicological data on REE-associated health effects are mostly confined to Ce and La, with less information for the other REEs.

Conducting further studies focusing on the potential bioaccumulation patterns of REEs in the environment and food and their health risks for humans is an urgent need, considering the increasing worldwide use of REEs in recent years and the scanty data on the dietary REE intake of humans. Moreover, it is necessary to assess the risk of REEs in foods in China to support any changes regarding the compulsory limit of REEs in food stipulated since the 1980s in China. The objectives of this study were (1) to investigate the concentrations and patterns of REEs in foods collected from 2011 to 2015 in China, and (2) to conduct a risk assessment of the dietary REEs exposure in the Chinese population.

## 2. Material and Methods

### 2.1. Sample and Preparation

A total of 27,457 individual food samples representing the major food categories were collected from supermarkets, local markets, and in the field in 31 provinces of China between 2011 and 2015. The food samples were aggregated into 11 main food categories that were further divided into subgroups. The main food categories included grains, vegetables, fruits, meat, aquatic products, milk, eggs, thallus, beans, nuts, and tea. The number of samples in each category can be found in Table 1. The samples were homogenized with a blender and stored in closed polyethylene vessels in a refrigerator at −20 °C.

### 2.2. Determination of REEs Concentrations in Foods

The REEs concentrations in the food samples were analyzed in local laboratories in 31 provinces of China. The same analysis procedure was used in all of the laboratories and training was provided before the analysis. The 16 REEs in foods were analyzed following a protocol for inductive coupled plasma-mass spectrometry (ICP-MS) elemental analysis in the China National Monitoring Handbook of Food Safety and China National Food Safety Standard GB 5009.94-2012 [22,23]. The microwave digestion system was applied, where 0.5 g of sample was weighed and digested with 5 mL concentrated nitric acid overnight. For animal derived foods, an additional 1 mL hydrogen peroxide solution was added during digestion. The digestion solution was then diluted to 10 mL with ultra-high purity water. Mixed solutions containing Rh, In, and Re were used as the online internal standard. Each sample was analyzed in duplicate.

Among the 17 REEs, Pm only occurs from the radioactive decay of Eu-151 or Uranium, which is radioactive without a stable isotope. Thus, 16 out of 17 REEs (except Pm) were determined in this study. The quality control was performed by the China National Center for Food Safety Risk Assessment. All of the laboratories that participated in the study were trained regarding uniform methodology and experimental techniques. Reference standards were added every 10 samples to perform extra tests for validation. Inter-laboratory validation was carried out for blind quality control samples and 2% of all food samples. The data of REEs concentrations were independently validated to ensure data quality and accuracy. The limits of detection (LOD) of the 16 REEs for the method ranged from 0.03 to 0.6 μg/kg for the different food categories.

### 2.3. Food Consumption Data

Food consumption data were obtained from the China Health and Nutrition Survey (CHNS) from 2010 to 2013 [24]. In this survey, a total of 61,811 study subjects were selected through stratified multi-stage cluster sampling from 31 provinces of China. Food consumption data were collected with a 24 h dietary recall method on three consecutive days at home. At the same time, the individual body weight was investigated. The food consumption within each food category was summed up for each investigated person to match with the REEs occurrence data of each food category.

### 2.4. REEs Health-Based Guidance Value

In 2016, the China Scientific Committee on Food Safety Risk Assessment set the temporary acceptable daily intake (tADI) of three main REEs, La (51.5 μg/kg BW), Ce (645.0 μg/kg BW), and Y (145.5 μg/kg BW), based on the No Observed Adverse Effect Level (NOAEL) of the critical effect in the decreased body weight gain from a 90 day feeding study in rats and the application of a safety factor of 200 for inter-species and intra-human variability [25,26]. In addition, the China Scientific Committee selected the lowest tADI of La as the health-based guidance value of the total REEs following the conservative principle of risk assessment, deriving a group tADI for the total REEs (51.5 μg/kg BW) [27]. Therefore, 51.5 μg/kg BW/d was used as the tADI of REEs in this study.

### 2.5. Estimation of REE Intake and Cumulative Risk Assessment

The daily mean intake of REE was calculated using Equation (1), multiplying the mean consumption data of each food category with the corresponding mean concentrations of REE for the food category, and adding it up to give the estimated total daily REE intake.

(1) $$\mathrm{EDI}=\frac{\sum_{j=1}^{n}{\;C}_{j}{\;\times \;F}_{j}}{\mathrm{BW}}$$

where *EDI* denotes the estimated daily dietary REE mean intake (μg/kg BW); *F_j_* denotes the daily mean consumption of food category *j* (g/day); *C_j_* denotes the mean concentration of REE in the corresponding food category *j* (mg/kg); *BW* refers to the mean body weight of the general population or each sub-group population (kg); and *n* refers to the number of food categories used to derive *EDI* for the general population or each sub-group population.

The exposure assessment model of the dietary REE exposure of the highly exposed consumers was undertaken in three steps according to EFSA guidance for the use of the concise database, assuming these consumers were highly exposed to two food categories, while they were exposed to an average level for the other categories [28]. The high exposure (the 95th percentile, P95) was calculated by adding the 95th percentile exposure of two food categories with the highest dietary contributions to consumers and the mean exposure levels of the other food categories for the general population.

The mean and the P95 exposures were obtained for the general population, and each subgroup, namely, toddlers (2–6 years), children (7–12 years), adolescents (13–17 years, male and female), and adults (over 18 years, male and female).

Additionally, the hazard index (*HI*) and the sum of the hazard quotient (*HQ*) of various chemicals were applied to assess the accumulative exposure of REEs (Equation (2)) [29].

(2) $$\mathrm{HI}=\sum_{i=1}^{n}{\mathrm{HQ}}_{i}=\sum_{i=1}^{n}\frac{{\mathrm{EDI}}_{i}}{RV_{i}}$$

where *HI* denotes the hazard index; *HQ_i_* denotes the hazard quotient of REE *i*; *EDI_i_* denoted the daily dietary intake of REE *i*; *RV_i_* denotes the health-based guidance value of REE *i*; and *n* refers to the number of REEs used to derive *HI* for the general population or each sub-group. Adverse health effects should be considered cautiously if *HI* > 1, whereas *HI* ≤ 1 suggests that the risk is acceptable.

### 2.6. Statistical Analysis

All statistical analyses were performed with R software 4.1.0. The mean and P95 of REEs exposure were examined and calculated for individual elements and the sum of all 16 REEs.

## 3. Results

### 3.1. Concentrations and Distributions of REEs in Foods

The detection rate of REEs in the food samples was 100%. As shown in Table 1 and Table S1, the mean and median concentration of the total REEs was 0.33 mg/kg and 0.02 mg/kg, respectively. The levels of REEs varied considerably among the food categories. The mean concentrations of the total REEs in different food categories ranged from 0.04–1.41 mg/kg. The mean concentration of the total REEs in tea was the highest (1.41 mg/kg), followed by thallus (1.14 mg/kg), eggs (0.29 mg/kg), and aquatic products (0.28 mg/kg). The mean concentrations in vegetables, fruits, grains, milk, meat, and beans were equal to or lower than 0.14 mg/kg.

**Table 1:** Concentrations of the total REEs in different food categories.

Food Categories	*n*	Concentrations of Total REEs (mg/kg)						
		Mean	P50	P90	P95	P97.5	P99	Maximum
Grains	4877	0.09	0.02	0.11	0.19	0.40	1.46	14.22
Vegetables	5192	0.14	0.02	0.21	0.42	0.86	1.96	43.93
Fruits	2860	0.04	0.01	0.08	0.16	0.32	0.45	2.27
Meat	5566	0.07	0.01	0.11	0.22	0.55	0.94	18.36
Aquatic products	3264	0.28	0.02	0.29	0.59	1.54	5.92	75.20
Milk	344	0.08	0.02	0.31	0.39	0.49	0.58	0.94
Eggs	598	0.29	0.02	0.69	1.48	2.94	5.75	8.81
Thallus	204	1.14	0.03	5.92	7.98	9.38	10.35	11.82
Beans	140	0.07	0.05	0.11	0.17	0.23	0.48	1.45
Nuts	83	0.10	0.05	0.10	0.11	0.13	0.14	1.45
Tea	4329	1.41	0.94	2.88	4.03	5.31	7.81	62.21
Total	27,457	0.33	0.02	0.89	1.67	2.69	4.39	75.20

**Figure 1:**
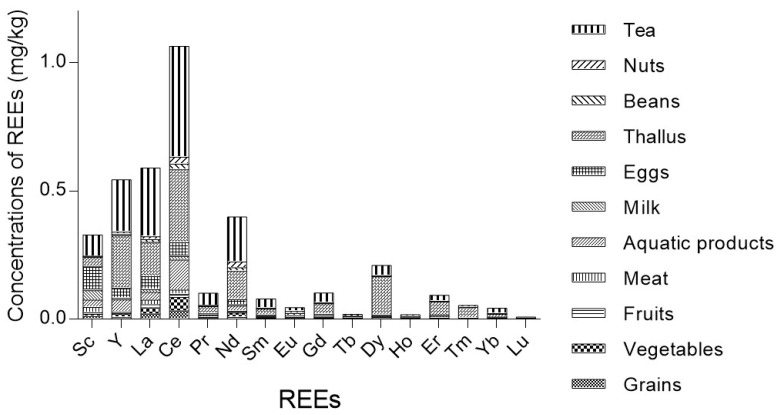
The distribution patterns of 16 REEs in different food categories.

In various food categories, the distribution patterns of 16 REEs were similar (Figure 1). Ce, La, Y, and Nd were the most abundant REEs. The mean concentrations of Ce, La, Y, and Nd in various food categories were higher than the other REEs, namely 0.11 mg/kg, 0.06 mg/kg, 0.05 mg/kg, and 0.04 mg/kg, respectively. Concentrations of Ce, La, and Y in different food categories were listed in Table S2, Table S3, and Table S4, respectively.

The sum of the mean concentrations of LREEs and HREEs for different food categories are shown in Table S5 and Table S6, respectively. The sum concentrations of the 6 LREEs and 10 HREEs for all food categories were 0.25 and 0.08 mg/kg, respectively. The mean concentrations of the total LREEs were higher than the total HREEs for all of the food categories, except for Cephalopoda, agaric, and kelp.

### 3.2. Estimation of Dietary REEs Exposures of the Chinese Population

#### 3.2.1. Dietary REEs Exposures by Different Sub-Groups

The dietary daily total REEs exposure for the general population and different sex-age groups are shown in Table 2. The daily mean exposure of the total REEs of the general population was 1.62 μg/kg BW, which was 3.14% of the tADI. The daily mean exposures of different sex–age groups were in the range of 1.61–2.80 μg/kg BW. Among the different sex–age groups, the mean exposure level of REEs in toddlers was the highest (2.80 μg/kg BW), but it was still only 5.44% of the tADI.

The P95 of the general population was 4.83 μg/kg BW, which was 9.38% of the tADI. The P95 of different sex–age groups ranged from 4.67 (male adults) to 8.22 (toddlers) μg/kg BW. The P95 of the general population and the sub-groups were all lower than the tADI.

The exposure levels of Ce, La, and Y among the 16 REEs were the highest. The daily mean exposure to these three REEs in the general population was 0.54, 0.31, and 0.17 μg/kg BW, respectively, and the sum of the exposures of these three REEs accounted for approximately 63% of the total exposure to the 16 REEs (Table 3).

The *HI* value of these three REEs in the mean and the P95 exposure of the general population was 0.008 and 0.022, respectively, far below 1 (Table 4). The *HI* value of the 16 REEs of the general population was also supposed to be far lower than 1, considering that the exposure to Ce, La, and Y accounted for approximately 63% of the total REEs exposure. Therefore, this indicated that the potential health risks posed by the dietary REEs intake in China was low.

#### 3.2.2. Contributions of Different Food Categories to REEs Exposure

**Figure 2:**
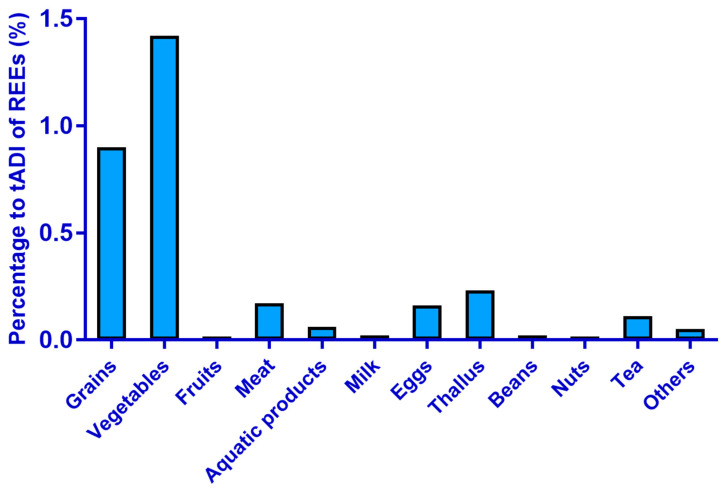
Percentage of mean total REEs exposure of different food categories to the tADI of REEs (51.5 μg/kg BW) for the general population.

The mean intake of the total REEs for the general population from any single food category did not reach 1.50% of the tADI (Figure 2).

**Figure 3:**
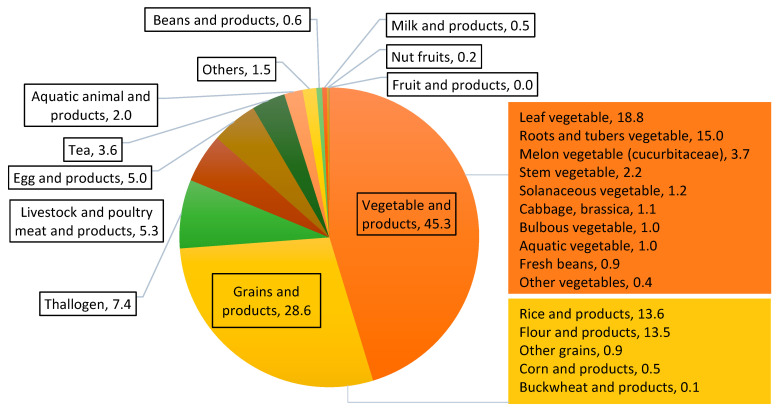
Contributions of different food categories to the exposure of total REEs in the general population (%).

Each food category was analyzed separately for the general population and different sex–age groups. No apparent differences were found for the contributions of different food categories to the total REEs exposure among sex–age groups. Therefore, only the contributions of different food categories for the general population are shown (Figure 3). The two most important contributors of dietary REEs intake were vegetables (45.3%) and grains (28.6%). In vegetables, leafy vegetables as well as root and tuber vegetables accounted for 18.8% and 15.0% of the REEs intake, respectively, mostly due to their higher consumption levels. In grains and grain-based products, the contribution rate of rice and wheat flour were the highest, namely 13.6% and 13.5%, respectively. In addition, the food categories with contribution rates higher than 5% included thallus (7.4%), meat (5.3%), and eggs (5.0%). The concentration of REEs in tea was the highest among all of the food categories, but the consumption of tea was low, resulting in a low contribution of tea (3.6%).

## 4. Discussions

This study determined the concentrations of 16 REEs in nationally representative food samples of 11 main food categories consumed in China. Combined with the food consumption data from a national survey, the REEs exposure level of the Chinese population was calculated. Our study suggested that the health risk from the dietary intake of REEs for the Chinese population was low. The major REEs consumed from food were La, Ce, and Y.

There have been several reports in the literature showing the concentrations of REEs in foods. In a survey in 2004, the range of the mean concentrations of 12 different food categories in four provinces of China was reported to be from less than 1 μg/kg to 40 μg/kg [30]. Wang et al. [31] collected 270 food samples such as cereals, meat, aquatic products, vegetables, fresh eggs, and tea from markets in Shanghai China, and reported that the detection rates of 16 REEs in foods ranged from 60.0% to 100.0%, the means of concentrations were 0.03–1.05 mg/kg in different food categories, and the highest mean concentration was detected in tea. Zhou et al. [32] demonstrated that the mean concentrations of 16 REEs in 12 food categories in China were 0.006–0.06 mg/kg from the Fourth China Total Diet Study. Dai et al. [33] collected 695 samples within 14 food categories from 33 cities in China, and reported that the concentration range of 13 REEs was 2.12–809.68 μg/kg with the highest mean concentration in shellfish. Yang et al. [34] reported the concentrations of REEs in freshwater and marine fish collected in the Shandong Province of China were in the range of 34.0–37.9 μg/kg and 12.7–37.6 μg/kg, respectively. Mayfield and Fairbrother [35] measured the concentrations of REEs in freshwater fish tissues in the United States, and the range was 0.014–3.0 mg/kg (dry weight). Mleczek et al. [36] found that the mean concentrations of REEs were 1.39 ± 1.21 mg/kg and 1.61 ± 0.97 mg/kg in 20 above-ground and growing on wood mushroom species (dry weight) in Poland, respectively. Squadrone et al. [37] reported the highest concentrations of REEs in lower trophic levels such as plant feed (1.8 mg/kg) and seaweeds (12 mg/kg), but several orders lower concentrations in higher trophic levels such as fish (0.21 mg/kg) in Northwestern Italy. Compared with the REEs concentrations in food samples from the literature, the concentration levels of REEs in our study were consistent with them.

The concentrations of REEs in this study varied considerably among the food categories. The result was consistent with the other study, e.g., it was reported that the concentrations of REEs in leafy and non-leafy vegetables were different [1]. The mean REEs concentration in tea was the highest. The high level of REEs in tea may be associated with the nature of the tea tree species themselves. It has been reported that the REE concentrations in plants are varied according to the contaminant levels in the environment and among different plant species [38]. REEs can be absorbed by the plants, including tea trees, via roots and leaves, and the REEs absorbed by the roots can eventually be translocated to the leaves, although apoplastic barriers exist [5,11]. The tea tree has been regarded as a plant that renders the absorption of REEs much easier [39].

In this study, nearly all of the food samples presented higher concentrations of LREEs than those of the HREEs; the mean ratio of the total LREEs to total HREEs was 3.01. Because of their higher mobility in soils, LREEs were easier to be absorbed by plants, thus presenting higher concentrations than the HREEs in plant tissues. In addition, as HREEs form much more stable complexes in the soil solution, the preferential absorption of LREEs was favored [5,40]. As animals eat plants as their foods, higher concentrations of LREEs can ultimately occur in animal-source foods. Similar results were demonstrated in other studies where LREEs had higher concentrations than HREEs [34,37]. In various food categories, the distribution patterns of 16 REEs were almost the same, and they were comparable to the natural crustal abundance [7]. Ce, La, Y, and Nd were the major elements of all REEs. The average abundances of these four REEs accounted for 76.67% of the total REEs.

Data on the dietary REEs intake of human beings are limited. In our study, the mean and P95 daily dietary REEs exposure of the general Chinese population was 1.62 and 4.83 μg/kg BW, respectively. In a survey in 2004, the mean daily REEs exposure of male adults in the four different dietary type areas of China was 55.9 μg (0.93 μg/kg BW based on 60 kg body weight) [30]. Wang et al. [31] reported that the mean and P95 dietary REEs intake of the general population were 108.73 and 245.00 μg/d (1.81 and 4.08 μg/kg BW), respectively, in Shanghai China. Zhou et al. [32] demonstrated that the mean daily REEs exposure of male adults in 12 provinces of China was 250.9 μg (4.18 μg/kg BW) from the Fourth China Total Diet Study. Dai et al. [33] reported that the mean dietary REEs intake was 0.54 μg/kg BW in China. Shi et al. [27] estimated that the dietary intake of REEs via fruits and vegetables in China were 0.02–0.06 μg/kg BW and 0.53–1.22 μg/kg BW, respectively. Compared with these reports of the daily dietary REEs exposure, the result using the total diet study method was higher than those of ours and other studies, which may be associated with the different methods of investigation. The total diet study method was carried out by aggregating the foods into groups with similarity and detecting the mixed samples.

The dietary intake of REEs by toddlers (2–6 years) was higher than other sex–age groups, indicating that the potential health risk for toddlers was higher than other sub-groups, athough the exposure still did not exceed the tADI. One possible reason for the higher intake in toddlers was that food consumption as a factor of body weight was greater in toddlers than in other sub-group populations.

For individual REEs, the mean daily dietary exposures of La, Ce, and Y in the general population were higher than the other REEs. The sum of these three REEs exposures accounted for approximately 63% of the total exposure to 16 REEs, that is, they contributed to most of the REEs exposure in dietary sources. In addition, the higher exposure levels of the three REEs were comparable to their high natural crustal abundance [7]. Similarly, Zhu et al. [30] reported that the dietary exposures of La and Ce were the highest among the 14 REEs. Zhou et al. [32] reported that the dietary exposures of Sc, Y, La, Ce, and Nd were higher than the other REEs, and the dietary exposure of different REEs varied in different regions of China.

*HI* was considered as a potential risk for adverse health effects from a mixture of chemicals to indicate the long-term risk assessment. The *HI* method has been used in many studies to assess the long-term risks of chemicals such as pesticide residues [41], plasticizers [42,43], and heavy metals [44]. In this study, the *HI* values were calculated for the cumulative exposure to La, Ce, and Y. As mentioned in Section 3.2.1, the *HI* values of these three REEs in the mean and the P95 exposure of the general population and sub-group population were all far below 1. The *HI* value of the total REEs was also supposed to be lower than 1, considering that exposure to La, Ce, and Y contributed most of the exposure. This result indicates that there were no severe potential health risks posed by dietary REEs intake in China.

The dietary contributions of REEs exposure were demonstrated in this study. Vegetables and grains were the two most important contributors, in accordance with Wang et al. [31] (vegetables = 28.6%, grains = 24.5%), Zhu et al. [30] (grains = 61.5%, vegetables = 22.6%), and Dai et al. [33] (grains 45.44–69.78%, followed by vegetable aquatic animal foods). The concentration of REEs in tea was the highest among all of the food categories, but with a lower consumption. Therefore, the contribution of tea to the dietary REEs exposure of the general population and the sub-group population was lower.

Up until now, the Codex Alimentarius Commission, the European Union, Australia, New Zealand, the USA, Canada, and Japan have not set the maximum limit for REEs in foods. The maximum limits for REEs in foods were set in the China Food Safety Standard from 1991 to 2017 (GB 2762-2012). The limit of REEs in rice, corn, wheat, and tea was 2.0 mg/kg, and the limit of REEs in vegetables and fruits was 0.7 mg/kg [45]. Our data showed that the REE concentrations in 1365 out of 27,457 food samples exceeded the maximum limit stipulated in GB2762-2012, and the over standard rate was 4.97%. According to our results, the actual contaminant level of REEs in food posed a low risk to the health of most Chinese people. Therefore, the limits stipulated in the standard were of little use to protect the health of consumers but increased the burden of the producers. Based on our results, Standard GB 2762 was amended in 2017 and the limits of REEs were removed [46].

There were some uncertainties in this study. First, not all of the foods were included in the analysis, despite the fact that the 11 main categories of foods consumed in China were surveyed. In addition, the food consumption data were collected more than 9 years ago, and the changes in Chinese dietary patterns were not taken into consideration in this study. Further assessment is recommended to determine whether the health risk of dietary REEs exposure in China changed by collecting new consumption data. Second, only exposure through foods was calculated. Additional exposure pathways such as cigarette smoking and inhalation exposure (e.g., PM_2.5_) may also play important roles for the human REE burden [47,48]. All of these uncertainties might influence the REE exposure of the Chinese population.

## 5. Conclusions

The health risk from dietary REEs exposure of the general Chinese population was within an acceptable level. The *HI* value of 16 REEs in the exposure of the general population was far lower than 1. The findings of this study provided scientific evidence supporting the decision of the National Health and Family Planning Commission of the People’s Republic of China to withdraw the limitations of REEs in foods from the China Food Safety Standard GB 2762 on 17 March 2017 [46].

## Figures and Tables

**Table 2:** Dietary mean and P95 exposure of total REEs estimated in the general population and different sex–age groups of the Chinese population (μg/kg BW).

Groups	*n*	Mean		P95	
		Exposure	% tADI *	Exposure	% tADI
2–6 years	4239	2.80	5.44	8.22	15.96
7–12 years	3256	2.23	4.33	6.95	13.49
13–17 years males	888	1.79	3.47	5.78	11.22
13–17 years females	824	1.72	3.34	5.89	11.43
≥18 years males	24,210	1.63	3.16	4.67	9.07
≥18 years females	28,394	1.61	3.13	4.82	9.37
Total	61,811	1.62	3.14	4.83	9.38

* %ADI represents the percentage of exposure to the tADI of REEs (51.5 μg/kg BW).

**Table 3:** Dietary mean and P95 exposure of Ce, La, and Y estimated in the general population and different sex–age groups of the Chinese population (μg/kg BW).

Groups	*n*	Ce				La				Y			
		Mean		P95		Mean		P95		Mean		P95	
		Exposure	% tADI *	Exposure	% tADI	Exposure	% tADI	Exposure	% tADI	Exposure	% tADI	Exposure	% tADI
2–6 years	4239	0.89	0.14	2.72	0.42	0.54	1.05	1.04	2.01	0.28	0.19	1.72	1.18
7–12 years	3256	0.74	0.11	1.85	0.29	0.43	0.83	0.93	1.80	0.22	0.15	1.22	0.84
13–17 years males	888	0.59	0.09	1.17	0.18	0.34	0.67	0.60	1.16	0.18	0.12	1.00	0.69
13–17 years females	824	0.57	0.09	1.57	0.24	0.33	0.64	0.63	1.22	0.17	0.12	0.78	0.54
≥18 years males	24,210	0.55	0.09	1.37	0.21	0.31	0.60	0.68	1.32	0.17	0.12	0.89	0.61
≥18 years females	28,394	0.54	0.08	1.48	0.23	0.30	0.59	0.74	1.43	0.17	0.11	1.03	0.71
Total	61,811	0.54	0.08	1.43	0.22	0.31	0.57	0.69	1.35	0.17	0.11	0.96	0.66

* %ADI represents the percentage of exposure to the tADI of Ce (645.0 μg/kg BW), La (51.5 μg/kg BW), and Y (145.5 μg/kg BW), respectively.

**Table 4:** The *HI* of the mean and P95 exposure of Ce, La, and Y in the general population and different sex–age groups of the Chinese population.

Groups	*n*	Mean Exposure *HI*	P95 Exposure *HI*
2–6 years	4239	0.014	0.036
7–12 years	3256	0.011	0.029
13–17 years males	888	0.009	0.020
13–17 years females	824	0.008	0.020
≥18 years males	24,210	0.008	0.021
≥18 years females	28,394	0.008	0.024
Total	61,811	0.008	0.022

## Data Availability

The China Health and Nutrition Survey (CHNS) data can be found at: https://www.cpc.unc.edu/projects/china.

## Supplementary Materials

The following supporting information can be downloaded at: https://www.mdpi.com/article/10.3390/ijerph192315583/s1, Table S1. Concentrations of total REEs in different food categories. Table S2. Concentrations of Ce in different food categories. Table S3. Concentrations of La in different food categories. Table S4. Concentrations of Y in different food categories. Table S5. The mean concentrations of 6 LREEs (La, Ce, Pr, Nd, Sm, Sc) in different food categories. Table S6. The mean concentrations of 10 HREEs (Eu, Gd, Tb, Dy, Ho, Er, Tm, Yb, Lu, Y) in different food categories. Table S7. The mean and P95 exposure of the 13 REEs (except Ce, La, and Y) in the general population and different sex-age groups of the Chinese population (μg/kg BW).
