# Supplementary material for: Dietary Exposure Assessment of Rare Earth Elements in the Chinese Population
Source: Int J Environ Res Public Health. 2022 Nov 24;19(23):15583. doi: 10.3390/ijerph192315583 (PMC9738814; doi:10.3390/ijerph192315583)
Supplement: Supplementary file 1 [file ijerph-19-15583-s001.zip › ijerph-2028142-supplementary.pdf]

Table S1. Concentrations of total REEs in different food categories.

Food categories	N	Concentrations of total REEs (mg/kg)						
		Mean	P50	P90	P95	P97.5	P99	Maximum
Grains	4877	0.09	0.02	0.11	0.19	0.40	1.46	14.22
Rice and rice products	1836	0.09	0.01	0.09	0.17	0.46	1.47	14.22
Flour and flour products	1072	0.11	0.03	0.14	0.24	0.52	1.97	7.24
Corn and corn products	1225	0.07	0.02	0.10	0.16	0.30	1.33	4.53
Buckwheat and buckwheat products	14	0.08	0.03	0.18	0.25	0.31	0.35	0.37
Other grains	730	0.07	0.02	0.12	0.19	0.38	0.69	4.39
Vegetables	5192	0.14	0.02	0.21	0.42	0.86	1.96	43.93
Bulbous vegetable	478	0.13	0.03	0.22	0.48	0.96	1.69	5.44
Stem vegetable	276	0.23	0.03	0.19	0.27	0.42	0.61	43.93
Brassica	494	0.09	0.01	0.10	0.27	0.57	1.69	6.90
Non-cucurbitaceae solanaceous vegetable	803	0.04	0.01	0.08	0.15	0.23	0.42	2.67
Leafy vegetable	1510	0.19	0.05	0.38	0.67	1.12	2.25	11.24
Fresh beans	327	0.04	0.02	0.09	0.14	0.24	0.45	1.12
Root and tuber vegetable	497	0.26	0.03	0.27	1.10	2.67	5.79	11.22
Cucurbitaceae	757	0.13	0.01	0.09	0.17	0.45	1.49	32.57
Aquatic vegetable	40	0.19	0.04	0.24	1.02	1.82	2.23	2.50
Other vegetables	10	0.06	0.03	0.08	0.23	0.31	0.36	0.39
Fruits	2860	0.04	0.01	0.08	0.16	0.32	0.45	2.27
Citrus	541	0.06	0.01	0.10	0.24	0.37	0.82	2.27
Pome	934	0.04	0.01	0.07	0.15	0.33	0.47	1.74
Stone fruit	383	0.03	0.01	0.06	0.13	0.22	0.40	0.69
Berry fruit	350	0.03	0.01	0.05	0.12	0.21	0.37	1.21
Melon fruit	171	0.03	0.01	0.08	0.13	0.28	0.41	0.81
Other fruits	562	0.04	0.01	0.08	0.17	0.31	0.49	1.78
Meat	5566	0.07	0.01	0.11	0.22	0.55	0.94	18.36

Food categories	N	Concentrations of total REEs (mg/kg)						
		Mean	P50	P90	P95	P97.5	P99	Maximum
Pork and pork products	1293	0.07	0.01	0.11	0.24	0.60	1.10	12.27
Beef and beef products	947	0.05	0.01	0.10	0.17	0.23	0.61	2.61
Mutton and mutton products	413	0.05	0.01	0.12	0.16	0.27	0.59	2.09
Chicken and chicken products	816	0.07	0.01	0.11	0.22	0.50	0.93	6.73
Other meat and products	12	0.01	0.00	0.02	0.02	0.02	0.02	0.02
Liver	1156	0.10	0.02	0.13	0.34	0.57	1.12	18.36
Kidney	929	0.08	0.01	0.11	0.34	0.57	0.75	16.13
Aquatic products	3264	0.28	0.02	0.29	0.59	1.54	5.92	75.20
Freshwater fishes	1678	0.21	0.02	0.20	0.33	0.66	6.33	18.25
Marine fishes	576	0.16	0.02	0.17	0.27	0.82	3.52	13.40
Freshwater shrimp	218	0.15	0.06	0.29	0.47	0.72	2.41	3.14
Marine shrimp	185	1.05	0.03	0.32	1.79	13.62	18.05	75.20
Freshwater crab	109	0.17	0.09	0.39	0.57	0.64	1.08	1.24
Marine crab	101	0.51	0.06	0.73	2.33	3.80	7.52	17.24
Bivalve	172	0.63	0.31	1.62	2.31	2.83	3.96	11.89
Cephalopoda	94	0.34	0.01	0.23	1.61	2.75	5.41	15.00
Other mollusc	47	0.39	0.06	0.77	1.04	1.87	5.83	9.08
Roe viscera	3	0.06	0.06	0.08	0.09	0.09	0.09	0.09
Milk	344	0.08	0.02	0.31	0.39	0.49	0.58	0.94
Milk powder	115	0.18	0.09	0.43	0.54	0.63	0.78	0.94
Liquid milk	229	0.03	0.01	0.07	0.12	0.17	0.26	0.33
Eggs	598	0.29	0.02	0.69	1.48	2.94	5.75	8.81
Fresh egg	415	0.21	0.01	0.48	1.45	1.76	3.08	6.88
Processed egg	183	0.47	0.03	0.94	3.26	6.17	8.29	8.81
Thallus	204	1.14	0.03	5.92	7.98	9.38	10.35	11.82
Agaric	19	2.05	0.06	7.20	8.37	9.74	10.56	11.11
Other fungus	126	1.06	0.03	3.82	7.88	9.48	10.29	11.82

Food categories	N	Concentrations of total REEs (mg/kg)						
		Mean	P50	P90	P95	P97.5	P99	Maximum
Nori	33	1.24	0.05	6.06	7.87	9.31	9.36	9.38
Kelp	24	0.70	0.03	0.61	5.84	7.27	7.70	7.99
Other algae	2	1.15	1.15	1.85	1.93	1.98	2.00	2.02
Beans	140	0.07	0.05	0.11	0.17	0.23	0.48	1.45
Soybean	61	0.05	0.04	0.09	0.10	0.12	0.17	0.23
Mung bean	40	0.07	0.04	0.13	0.23	0.30	0.48	0.60
Azuki bean	39	0.10	0.05	0.16	0.17	0.28	0.98	1.45
Nuts	83	0.10	0.05	0.10	0.11	0.13	0.14	1.45
Tea	4329	1.41	0.94	2.88	4.03	5.31	7.81	62.21
Total	27457	0.33	0.02	0.89	1.67	2.69	4.39	75.20

Table S2. Concentrations of Ce in different food categories.

Food categories	N	Concentrations of Ce (mg/kg)						
		Mean	P50	Mean	P95	Mean	P99	Range
Grains	4877	0.0326	0.0027	0.0289	0.0593	0.1440	0.5008	0.0000-6.3400
Rice and rice products	1836	0.0331	0.0013	0.0244	0.0552	0.1512	0.5111	0.0000-6.3400
Flour and flour products	1072	0.0419	0.0075	0.0450	0.0816	0.1472	0.5413	0.0000-5.8100
Corn and corn products	1225	0.0238	0.0028	0.0230	0.0470	0.1152	0.3832	0.0000-3.5600
Buckwheat and buckwheat products	14	0.0107	0.0038	0.0308	0.0350	0.0379	0.0396	0.0001-0.0407
Other grains	730	0.0330	0.0037	0.0240	0.0489	0.1300	0.4182	0.0000-3.7800
Vegetables	5192	0.0533	0.0050	0.0720	0.1439	0.3227	0.7491	0.0000-43.9000
Bulbous vegetable	478	0.0434	0.0068	0.0762	0.1742	0.4033	0.6568	0.0000-1.7683
Stem vegetable	276	0.1835	0.0074	0.0595	0.0827	0.1408	0.4418	0.0000-43.9000
Brassica	494	0.0326	0.0022	0.0237	0.0956	0.2088	0.7078	0.0000-4.2228
Non-cucurbitaceae solanaceous vegetable	803	0.0097	0.0019	0.0166	0.0319	0.0719	0.1398	0.0000-1.0050
Leafy vegetable	1510	0.0701	0.0130	0.1280	0.2443	0.4187	0.8791	0.0000-5.6100
Fresh beans	327	0.0128	0.0050	0.0255	0.0388	0.0801	0.1470	0.0000-0.4050
Root and tuber vegetable	497	0.0729	0.0050	0.0895	0.3882	0.8368	1.6440	0.0000-2.6400
Cucurbitaceae	757	0.0422	0.0029	0.0251	0.0571	0.1536	0.5360	0.0000-14.0322
Aquatic vegetable	40	0.0762	0.0093	0.0841	0.4332	0.7495	0.9478	0.0001-1.0800
Other vegetables	10	0.0428	0.0040	0.0581	0.2091	0.2845	0.3298	0.0016-0.3600
Fruits	2860	0.0102	0.0014	0.0144	0.0384	0.0705	0.1920	0.0000-1.5500
Citrus	541	0.0145	0.0018	0.0179	0.0455	0.1115	0.2618	0.0001-0.7980
Pome	934	0.0097	0.0012	0.0165	0.0378	0.0599	0.1538	0.0000-1.5500
Stone fruit	383	0.0086	0.0015	0.0124	0.0316	0.0469	0.1859	0.0000-0.4350
Berry fruit	350	0.0073	0.0004	0.0074	0.0269	0.0713	0.1310	0.0000-0.3900
Melon fruit	171	0.0155	0.0017	0.0137	0.0484	0.1387	0.2820	0.0001-0.7980
Other fruits	562	0.0089	0.0015	0.0110	0.0310	0.0572	0.1590	0.0000-0.5810
Meat	5566	0.0179	0.0010	0.0140	0.0304	0.0670	0.3012	0.0000-8.8400

Food categories	N	Concentrations of Ce (mg/kg)						
		Mean	P50	Mean	P95	Mean	P99	Range
Pork and pork products	1293	0.0171	0.0004	0.0110	0.0240	0.0624	0.4469	0.0000-4.2900
Beef and beef products	947	0.0100	0.0008	0.0100	0.0184	0.0369	0.1340	0.0000-1.3700
Mutton and mutton products	413	0.0052	0.0008	0.0110	0.0216	0.0436	0.0688	0.0001-0.2780
Chicken and chicken products	816	0.0238	0.0008	0.0144	0.0278	0.0541	0.7043	0.0001-2.4200
Other meat and products	12	0.0014	0.0007	0.0037	0.0053	0.0061	0.0066	0.0000-0.0070
Liver	1156	0.0241	0.0020	0.0205	0.0410	0.0824	0.3224	0.0001-8.8400
Kidney	929	0.0202	0.0011	0.0154	0.0560	0.0824	0.2338	0.0000-4.6648
Aquatic products	3264	0.1167	0.0024	0.0686	0.1768	0.4207	1.4678	0.0000-16.4000
Freshwater fishes	1678	0.1146	0.0015	0.0182	0.0650	0.1582	1.7725	0.0000-16.0000
Marine fishes	576	0.0435	0.0015	0.0260	0.0578	0.2806	0.9762	0.0001-5.3671
Freshwater shrimp	218	0.0448	0.0127	0.1022	0.1620	0.2763	0.4185	0.0000-0.9587
Marine shrimp	185	0.4151	0.0062	0.1328	0.4720	6.5098	12.9120	0.0000-16.4000
Freshwater crab	109	0.0446	0.0227	0.1122	0.1378	0.2308	0.2899	0.0001-0.4070
Marine crab	101	0.2764	0.0170	0.2200	0.9824	1.1657	2.7952	0.0000-15.8000
Bivalve	172	0.1935	0.0791	0.4578	0.6990	0.9430	1.0729	0.0000-4.7400
Cephalopoda	94	0.0382	0.0014	0.0331	0.2663	0.4112	0.7106	0.0001-1.0463
Other mollusc	47	0.1274	0.0134	0.1280	0.2983	0.6339	2.2936	0.0000-3.6587
Roe viscera	3	0.0155	0.0174	0.0247	0.0256	0.0260	0.0263	0.0025-0.0265
Milk	344	0.0142	0.0008	0.0254	0.0724	0.1663	0.3023	0.0001-0.5830
Milk powder	115	0.0308	0.0046	0.0838	0.1659	0.2345	0.3155	0.0001-0.5830
Liquid milk	229	0.0059	0.0006	0.0035	0.0088	0.0317	0.2075	0.0001-0.3230
Eggs	598	0.0539	0.0008	0.0199	0.1299	0.5492	1.4223	0.0000-5.3419
Fresh egg	415	0.0249	0.0002	0.0160	0.0529	0.2300	0.5701	0.0000-2.9100
Processed egg	183	0.1197	0.0028	0.0546	0.5802	1.4588	2.1526	0.0001-5.3419
Thallus	204	0.2834	0.0047	0.7670	1.3403	3.3289	4.4632	0.0000-4.8822
Agaric	19	0.5225	0.0070	1.1186	2.7141	3.7981	4.4485	0.0000-4.8822
Other fungus	126	0.2778	0.0045	0.7151	1.2480	3.6844	4.3406	0.0000-4.5802

Food categories	N	Concentrations of Ce (mg/kg)						
		Mean	P50	Mean	P95	Mean	P99	Range
Nori	33	0.2777	0.0096	0.8834	1.7002	2.3984	2.6525	0.0002-2.8218
Kelp	24	0.1214	0.0034	0.2395	0.8992	1.1494	1.2655	0.0002-1.3428
Other algae	2	0.4015	0.4015	0.6403	0.6701	0.6851	0.6940	0.1030-0.7000
Beans	140	0.0224	0.0144	0.0569	0.0856	0.0945	0.0956	0.0002-0.1123
Soybean	61	0.0205	0.0124	0.0438	0.0669	0.0857	0.0948	0.0002-0.0952
Mung bean	40	0.0206	0.0116	0.0455	0.0803	0.0941	0.105	0.0002-0.1123
Azuki bean	39	0.0271	0.0181	0.0815	0.0927	0.0946	0.0953	0.0003-0.0958
Nuts	83	0.0271	0.0160	0.0577	0.0697	0.0752	0.0874	0.0003-0.0958
Tea	4329	0.4315	0.2657	0.8643	1.2384	1.5576	2.0672	0.0000-58.1937
Total	27457	0.1061	0.0035	0.2558	0.5080	0.8211	1.3643	0.0000-58.1937

Table S3. Concentrations of La in different food categories.

Food categories	N	Concentrations of La (mg/kg)						
		Mean	P50	P90	P95	P97.5	P99	Range
Grains	4877	0.0204	0.0025	0.0252	0.0542	0.0951	0.3092	0.0000-3.9700
Rice and rice products	1836	0.0159	0.0018	0.0201	0.0437	0.0834	0.2755	0.0000-2.3621
Flour and flour products	1072	0.0282	0.0037	0.033	0.0587	0.1119	0.4304	0.0000-3.9700
Corn and corn products	1225	0.0235	0.0025	0.023	0.0466	0.0766	0.2576	0.0000-3.8000
Buckwheat and buckwheat products	14	0.0129	0.0027	0.0376	0.0504	0.0568	0.0607	0.0002-0.0633
Other grains	730	0.0148	0.0025	0.028	0.0731	0.1081	0.2031	0.0002-0.6010
Vegetables	5192	0.0242	0.0029	0.0436	0.088	0.1634	0.387	0.0000-5.3859
Bulbous vegetable	478	0.0205	0.0037	0.0411	0.0804	0.145	0.3297	0.0000-0.9032
Stem vegetable	276	0.014	0.0044	0.0315	0.0424	0.0732	0.1225	0.0000-0.4730
Brassica	494	0.0155	0.0015	0.0184	0.0657	0.1146	0.388	0.0000-0.6910
Non-cucurbitaceae solanaceous vegetable	803	0.0083	0.0012	0.0121	0.0307	0.0698	0.1403	0.0000-0.5000
Leafy vegetable	1510	0.0344	0.008	0.0781	0.1211	0.1976	0.4081	0.0000-1.7600
Fresh beans	327	0.0086	0.0029	0.017	0.0285	0.0531	0.0956	0.0000-0.2820
Root and tuber vegetable	497	0.0435	0.004	0.061	0.2692	0.4672	0.8368	0.0000-1.4700
Cucurbitaceae	757	0.0259	0.002	0.0196	0.0446	0.091	0.2935	0.0000-5.3859
Aquatic vegetable	40	0.0429	0.0072	0.0569	0.2339	0.4072	0.5055	0.0002-0.5710
Other vegetables	10	0.0042	0.0028	0.01	0.011	0.0115	0.0118	0.0000-0.0120
Fruits	2860	0.0116	0.001	0.014	0.0353	0.0661	0.1751	0.0000-2.2200
Citrus	541	0.0229	0.0018	0.025	0.0555	0.131	0.3398	0.0001-2.2200
Pome	934	0.0097	0.0011	0.014	0.0319	0.0513	0.1604	0.0000-0.9330
Stone fruit	383	0.007	0.0008	0.0131	0.0296	0.051	0.1384	0.0000-0.3400
Berry fruit	350	0.0068	0.0005	0.011	0.0332	0.054	0.0937	0.0000-0.4460
Melon fruit	171	0.0061	0.0011	0.0101	0.0216	0.0369	0.0887	0.0002-0.2630
Other fruits	562	0.0118	0.001	0.0117	0.0338	0.0652	0.1829	0.0000-1.7600
Meat	5566	0.0187	0.0008	0.0142	0.039	0.0824	0.3409	0.0000-12.1000

Food categories	N	Concentrations of La (mg/kg)						
		Mean	P50	P90	P95	P97.5	P99	Range
Pork and pork products	1293	0.0158	0.0005	0.01	0.0301	0.0808	0.3505	0.0000-3.3000
Beef and beef products	947	0.0083	0.0007	0.0101	0.0283	0.0498	0.1208	0.0000-1.1100
Mutton and mutton products	413	0.0167	0.0007	0.0139	0.047	0.08	0.1752	0.0002-2.0500
Chicken and chicken products	816	0.0163	0.0007	0.0144	0.0333	0.0715	0.3356	0.0000-4.2100
Other meat and products	12	0.0015	0.0004	0.0026	0.0064	0.0086	0.0099	0.0000-0.0108
Liver	1156	0.0338	0.0014	0.0206	0.0512	0.1331	0.5489	0.0000-12.1000
Kidney	929	0.0176	0.0011	0.0216	0.042	0.0777	0.2299	0.0000-2.7740
Aquatic products	3264	0.0298	0.0018	0.0432	0.124	0.2431	0.4889	0.0000-3.1554
Freshwater fishes	1678	0.0203	0.0012	0.0226	0.0647	0.163	0.3412	0.0000-3.0263
Marine fishes	576	0.0208	0.001	0.0188	0.0338	0.1996	0.422	0.0001-2.2456
Freshwater shrimp	218	0.0229	0.0067	0.0457	0.0722	0.1748	0.2425	0.0000-0.6052
Marine shrimp	185	0.0547	0.0023	0.0418	0.1774	0.3188	1.2946	0.0000-3.0571
Freshwater crab	109	0.0277	0.0166	0.0654	0.0963	0.11	0.133	0.0001-0.3400
Marine crab	101	0.0576	0.01	0.0845	0.308	0.4964	0.61	0.0000-1.3702
Bivalve	172	0.1165	0.0458	0.308	0.4302	0.537	0.5784	0.0000-2.3800
Cephalopoda	94	0.0204	0.0002	0.0157	0.1156	0.2339	0.341	0.0002-0.6030
Other mollusc	47	0.0961	0.0076	0.0801	0.158	0.3504	1.8797	0.0000-3.1554
Roe viscera	3	0.0091	0.0099	0.0104	0.0104	0.0105	0.0105	0.0070-0.0105
Milk	344	0.0105	0.0015	0.0281	0.0488	0.0853	0.1334	0.0002-0.1500
Milk powder	115	0.0206	0.0081	0.0568	0.103	0.1309	0.148	0.0002-0.1500
Liquid milk	229	0.0054	0.0007	0.0119	0.0281	0.0463	0.0631	0.0002-0.1480
Eggs	598	0.051	0.0017	0.0443	0.1344	0.5865	1.0886	0.0000-3.7500
Fresh egg	415	0.0508	0.0012	0.0167	0.1034	0.3306	1.4574	0.0000-3.7500
Processed egg	183	0.0513	0.0035	0.0946	0.254	0.7632	0.8838	0.0000-1.0796
Thallus	204	0.1337	0.0036	0.4952	0.7474	1.1648	1.4286	0.0000-1.8666
Agaric	19	0.1803	0.0056	0.6259	0.746	0.8204	0.865	0.0000-0.8948
Other fungus	126	0.1297	0.0037	0.4108	0.7322	1.3269	1.5724	0.0000-1.8666

Food categories	N	Concentrations of La (mg/kg)						
		Mean	P50	P90	P95	P97.5	P99	Range
Nori	33	0.162	0.0093	0.6379	1.0515	1.2037	1.2581	0.0002-1.2944
Kelp	24	0.0716	0.0024	0.0996	0.5767	0.7148	0.7612	0.0000-0.7921
Other algae	2	0.2202	0.2202	0.3528	0.3694	0.3777	0.3827	0.0544-0.3860
Beans	140	0.0108	0.0092	0.0184	0.0262	0.0325	0.0394	0.0002-0.0620
Soybean	61	0.0101	0.0083	0.0151	0.0211	0.0253	0.0312	0.0006-0.0387
Mung bean	40	0.0115	0.0075	0.0224	0.034	0.0405	0.0534	0.0002-0.0620
Azuki bean	39	0.0112	0.0109	0.0171	0.0243	0.0291	0.0304	0.0010-0.0312
Nuts	83	0.0112	0.0095	0.0167	0.0188	0.0233	0.028	0.0010-0.0312
Tea	4329	0.2673	0.177	0.5696	0.7806	1.0729	1.4164	0.0000-16.8000
Total	27457	0.0612	0.0026	0.1698	0.3233	0.5163	0.8388	0.0000-16.8000

Table S4. Concentrations of Y in different food categories.

Food categories	N	Concentrations of Y (mg/kg)						
		Mean	P50	P90	Mean	P97.5	P99	Range
Grains	4877	0.0068	0.0013	0.0080	0.0162	0.0272	0.0624	0.0000-3.6756
Rice and rice products	1836	0.0097	0.0007	0.0059	0.0122	0.0278	0.0952	0.0000-3.6756
Flour and flour products	1072	0.0077	0.0024	0.0161	0.0237	0.0344	0.0747	0.0000-1.3900
Corn and corn products	1225	0.0040	0.0014	0.0065	0.0126	0.0199	0.0432	0.0000-0.3870
Buckwheat and buckwheat products	14	0.0073	0.0046	0.0154	0.0195	0.0227	0.0246	0.0001-0.0259
Other grains	730	0.0030	0.0012	0.0057	0.0120	0.0194	0.0330	0.0001-0.0840
Vegetables	5192	0.0137	0.0014	0.0160	0.0382	0.0749	0.2471	0.0000-2.6812
Bulbous vegetable	478	0.0160	0.0019	0.0192	0.0394	0.0908	0.2200	0.0000-2.0982
Stem vegetable	276	0.0059	0.0023	0.0150	0.0214	0.0299	0.0491	0.0000-0.1730
Brassica	494	0.0084	0.0007	0.0061	0.0219	0.0539	0.2209	0.0000-0.5851
Non-cucurbitaceae solanaceous vegetable	803	0.0026	0.0006	0.0050	0.0068	0.0142	0.0387	0.0000-0.2600
Leafy vegetable	1510	0.0194	0.0030	0.0337	0.0592	0.1065	0.2613	0.0000-2.6293
Fresh beans	327	0.0036	0.0015	0.0069	0.0101	0.0194	0.0544	0.0000-0.1020
Root and tuber vegetable	497	0.0260	0.0015	0.0183	0.1200	0.2920	0.5773	0.0000-1.9500
Cucurbitaceae	757	0.0145	0.0007	0.0053	0.0096	0.0409	0.2072	0.0000-2.6812
Aquatic vegetable	40	0.0250	0.0027	0.0292	0.2109	0.2682	0.2957	0.0001-0.3140
Other vegetables	10	0.0029	0.0007	0.0045	0.0122	0.0161	0.0184	0.0000-0.0199
Fruits	2860	0.0023	0.0006	0.0038	0.0076	0.0146	0.0290	0.0000-0.3610
Citrus	541	0.0036	0.0007	0.0050	0.0082	0.0161	0.0444	0.0001-0.3610
Pome	934	0.0020	0.0006	0.0037	0.0076	0.0139	0.0287	0.0000-0.0682
Stone fruit	383	0.0020	0.0006	0.0048	0.0077	0.0141	0.0290	0.0000-0.0490
Berry fruit	350	0.0026	0.0003	0.0025	0.0063	0.0098	0.0238	0.0000-0.2400
Melon fruit	171	0.0023	0.0005	0.0030	0.0055	0.0152	0.0303	0.0001-0.1160
Other fruits	562	0.0019	0.0006	0.0034	0.0059	0.0140	0.0274	0.0000-0.0900
Meat	5566	0.0049	0.0003	0.0030	0.0091	0.0360	0.0768	0.0000-2.7349

Food categories	N	Concentrations of Y (mg/kg)						
		Mean	P50	P90	Mean	P97.5	P99	Range
Pork and pork products	1293	0.0046	0.0002	0.0027	0.0088	0.0370	0.0705	0.0000-1.4900
Beef and beef products	947	0.0026	0.0003	0.0025	0.0070	0.0210	0.0345	0.0000-0.6540
Mutton and mutton products	413	0.0020	0.0002	0.0025	0.0079	0.0198	0.0368	0.0000-0.0640
Chicken and chicken products	816	0.0047	0.0003	0.0031	0.0095	0.0330	0.0425	0.0000-0.8700
Other meat and products	12	0.0007	0.0002	0.0010	0.0026	0.0035	0.0041	0.0001-0.0045
Liver	1156	0.0055	0.0004	0.0034	0.0095	0.0641	0.0865	0.0000-1.4182
Kidney	929	0.0085	0.0004	0.0038	0.0230	0.0690	0.0880	0.0000-2.7349
Aquatic products	3264	0.0474	0.0009	0.0227	0.0641	0.1655	0.3541	0.0000-74.8000
Freshwater fishes	1678	0.0108	0.0006	0.0038	0.0140	0.0362	0.1650	0.0000-2.8321
Marine fishes	576	0.0326	0.0005	0.0068	0.0200	0.0494	0.3725	0.0000-6.0100
Freshwater shrimp	218	0.0203	0.0043	0.0288	0.0513	0.0802	0.3516	0.0000-1.3233
Marine shrimp	185	0.4453	0.0019	0.0284	0.1740	0.4259	1.7926	0.0000-74.8000
Freshwater crab	109	0.0213	0.0137	0.0533	0.0654	0.0685	0.1079	0.0001-0.2060
Marine crab	101	0.0521	0.0065	0.0512	0.2890	0.5839	0.9080	0.0000-1.1040
Bivalve	172	0.0874	0.0412	0.2263	0.2997	0.3541	0.7975	0.0000-1.6071
Cephalopoda	94	0.0688	0.0006	0.0168	0.1442	0.4103	2.4699	0.0001-2.6200
Other mollusc	47	0.0373	0.0041	0.0659	0.1590	0.3232	0.4222	0.0000-0.4837
Roe viscera	3	0.0072	0.0077	0.0124	0.0130	0.0133	0.0135	0.0004-0.0136
Milk	344	0.0061	0.0005	0.0181	0.0427	0.0569	0.0733	0.0001-0.1460
Milk powder	115	0.0161	0.0029	0.0516	0.0625	0.0787	0.0976	0.0001-0.1460
Liquid milk	229	0.0010	0.0002	0.0010	0.0021	0.0037	0.0272	0.0001-0.0470
Eggs	598	0.0391	0.0002	0.0122	0.0542	0.1966	1.3224	0.0000-3.5156
Fresh egg	415	0.0145	0.0002	0.0076	0.0294	0.0850	0.2957	0.0000-1.4000
Processed egg	183	0.0948	0.0009	0.0262	0.2366	1.2578	3.3220	0.0000-3.5156
Thallus	204	0.2049	0.0015	0.4747	1.2230	2.7123	3.7784	0.0000-4.9953
Agaric	19	0.1442	0.0025	0.5456	0.6615	0.8661	0.9889	0.0000-1.0707
Other fungus	126	0.1647	0.0015	0.3007	0.9813	1.5359	2.9085	0.0000-4.9953

Food categories	N	Concentrations of Y (mg/kg)						
		Mean	P50	P90	Mean	P97.5	P99	Range
Nori	33	0.3642	0.0015	0.8219	2.7063	3.9068	4.1729	0.0001-4.3503
Kelp	24	0.2524	0.0010	0.0697	2.3677	2.9176	3.0368	0.0001-3.1163
Other algae	2	0.1199	0.1199	0.1880	0.1965	0.2007	0.2033	0.0348-0.2050
Beans	140	0.0058	0.0042	0.0098	0.0154	0.0243	0.0383	0.0002-0.0784
Soybean	61	0.0045	0.0038	0.0075	0.01	0.0122	0.0165	0.0002-0.0200
Mung bean	40	0.0053	0.0032	0.0102	0.0179	0.0226	0.0257	0.0002-0.0277
Azuki bean	39	0.0085	0.0051	0.0115	0.0278	0.0468	0.0657	0.0008-0.0784
Nuts	83	0.0085	0.0043	0.0081	0.0092	0.0106	0.0113	0.0008-0.0784
Tea	4329	0.2044	0.1124	0.4508	0.6200	0.8516	1.3885	0.0000-5.9000
Total	27457	0.0454	0.0012	0.0990	0.2296	0.3800	0.6389	0.0000-74.8000

Table S5. The mean concentrations of 6 LREEs (La, Ce, Pr, Nd, Sm, Sc) in different food categories.

Food categories	Concentrations (mg/kg)						
	La	Ce	Pr	Nd	Sm	Sc	Total
Grains	0.020	0.033	0.002	0.008	0.002	0.010	0.075
Rice and rice products	0.016	0.033	0.003	0.010	0.002	0.010	0.073
Flour and flour products	0.028	0.042	0.003	0.008	0.002	0.013	0.096
Corn and corn products	0.024	0.024	0.001	0.005	0.001	0.009	0.064
Buckwheat and buckwheat products	0.013	0.011	0.003	0.005	0.003	0.031	0.066
Other grains	0.015	0.033	0.001	0.006	0.001	0.011	0.066
Vegetables	0.024	0.053	0.004	0.017	0.003	0.009	0.110
Bulbous vegetable	0.021	0.043	0.004	0.016	0.003	0.010	0.097
Stem vegetable	0.014	0.184	0.002	0.009	0.002	0.007	0.218
Brassica	0.016	0.033	0.002	0.010	0.002	0.008	0.070
Non-cucurbitaceae solanaceous vegetable	0.008	0.010	0.001	0.004	0.001	0.005	0.029
Leafy vegetable	0.034	0.070	0.007	0.026	0.005	0.013	0.156
Fresh beans	0.009	0.013	0.001	0.005	0.001	0.006	0.035
Root and tuber vegetable	0.044	0.073	0.006	0.029	0.004	0.006	0.161
Cucurbitaceae	0.026	0.042	0.004	0.016	0.003	0.009	0.100
Aquatic vegetable	0.043	0.076	0.005	0.028	0.003	0.004	0.158
Other vegetables	0.004	0.043	0.001	0.003	0.001	0.004	0.055
Fruits	0.012	0.010	0.001	0.002	0.001	0.007	0.032
Citrus	0.023	0.015	0.001	0.003	0.001	0.007	0.049
Pome	0.010	0.010	0.001	0.002	0.001	0.006	0.029
Stone fruit	0.007	0.009	0.001	0.002	0.001	0.004	0.022
Berry fruit	0.007	0.007	0.001	0.002	0.000	0.005	0.022
Melon fruit	0.006	0.016	0.001	0.001	0.000	0.004	0.028
Other fruits	0.012	0.009	0.001	0.002	0.001	0.011	0.034
Meat	0.019	0.018	0.001	0.005	0.001	0.020	0.064

Food categories	Concentrations (mg/kg)						
	La	Ce	Pr	Nd	Sm	Sc	Total
Pork and pork products	0.016	0.017	0.001	0.007	0.001	0.018	0.060
Beef and beef products	0.008	0.010	0.001	0.002	0.001	0.018	0.039
Mutton and mutton products	0.017	0.005	0.001	0.002	0.001	0.022	0.047
Chicken and chicken products	0.016	0.024	0.001	0.004	0.001	0.021	0.066
Other meat and products	0.002	0.001	0.000	0.001	0.000	0.002	0.006
Liver	0.034	0.024	0.002	0.008	0.001	0.021	0.090
Kidney	0.018	0.020	0.002	0.007	0.001	0.022	0.070
Aquatic products	0.030	0.117	0.006	0.020	0.004	0.029	0.206
Freshwater fishes	0.020	0.115	0.003	0.010	0.002	0.037	0.187
Marine fishes	0.021	0.044	0.007	0.016	0.004	0.018	0.109
Freshwater shrimp	0.023	0.045	0.004	0.016	0.004	0.025	0.118
Marine shrimp	0.055	0.415	0.015	0.047	0.009	0.028	0.569
Freshwater crab	0.028	0.045	0.010	0.016	0.004	0.021	0.123
Marine crab	0.058	0.276	0.013	0.046	0.011	0.017	0.420
Bivalve	0.117	0.194	0.024	0.092	0.020	0.023	0.468
Cephalopoda	0.020	0.038	0.006	0.019	0.004	0.007	0.094
Other mollusc	0.096	0.127	0.013	0.047	0.008	0.038	0.329
Roe viscera	0.009	0.016	0.002	0.008	0.002	0.005	0.041
Milk	0.011	0.014	0.002	0.005	0.001	0.037	0.070
Milk powder	0.021	0.031	0.005	0.013	0.003	0.082	0.155
Liquid milk	0.005	0.006	0.000	0.001	0.000	0.014	0.027
Eggs	0.051	0.054	0.005	0.020	0.005	0.094	0.228
Fresh egg	0.051	0.025	0.003	0.010	0.002	0.094	0.185
Processed egg	0.051	0.120	0.010	0.043	0.010	0.092	0.327
Thallus	0.134	0.283	0.028	0.110	0.024	0.036	0.615
Agaric	0.180	0.523	0.028	0.116	0.020	0.057	0.923
Other fungus	0.130	0.278	0.026	0.099	0.021	0.035	0.588

Food categories	Concentrations (mg/kg)						
	La	Ce	Pr	Nd	Sm	Sc	Total
Nori	0.162	0.278	0.039	0.157	0.038	0.039	0.712
Kelp	0.072	0.121	0.021	0.090	0.022	0.021	0.346
Other algae	0.220	0.402	0.048	0.176	0.032	0.063	0.940
Beans	0.011	0.022	0.002	0.014	0.002	0.002	0.053
Soybean	0.010	0.021	0.002	0.007	0.002	0.002	0.043
Mung bean	0.012	0.021	0.002	0.016	0.001	0.002	0.053
Azuki bean	0.011	0.027	0.002	0.023	0.001	0.002	0.067
Nuts	0.011	0.027	0.002	0.023	0.001	0.002	0.067
Tea	0.267	0.432	0.048	0.176	0.036	0.084	1.043
Total	0.061	0.106	0.010	0.037	0.008	0.028	0.250

Table S6. The mean concentrations of 10 HREEs (Eu, Gd, Tb, Dy, Ho, Er, Tm, Yb, Lu, Y) in different food categories.

Food categories	Concentrations (mg/kg)										
	Eu	Gd	Tb	Dy	Ho	Er	Tm	Yb	Lu	Y	Total
Grains	0.001	0.002	0.000	0.001	0.000	0.001	0.000	0.001	0.000	0.007	0.013
Rice and rice products	0.001	0.002	0.000	0.001	0.000	0.001	0.000	0.001	0.000	0.010	0.017
Flour and flour products	0.001	0.002	0.000	0.001	0.000	0.001	0.000	0.001	0.000	0.008	0.014
Corn and corn products	0.001	0.001	0.000	0.001	0.000	0.001	0.000	0.001	0.000	0.004	0.008
Buckwheat and buckwheat products	0.002	0.002	0.001	0.001	0.000	0.001	0.000	0.000	0.000	0.007	0.014
Other grains	0.001	0.001	0.000	0.001	0.000	0.000	0.000	0.000	0.000	0.003	0.007
Vegetables	0.001	0.003	0.001	0.005	0.001	0.003	0.002	0.001	0.001	0.014	0.031
Bulbous vegetable	0.001	0.003	0.001	0.002	0.001	0.002	0.001	0.002	0.000	0.016	0.029
Stem vegetable	0.001	0.002	0.000	0.001	0.000	0.001	0.000	0.001	0.000	0.006	0.012
Brassica	0.001	0.002	0.001	0.001	0.001	0.001	0.000	0.001	0.000	0.008	0.016
Non-cucurbitaceae solanaceous vegetable	0.001	0.001	0.001	0.001	0.001	0.001	0.001	0.000	0.001	0.003	0.009
Leafy vegetable	0.001	0.005	0.001	0.004	0.001	0.002	0.001	0.002	0.001	0.019	0.036
Fresh beans	0.001	0.001	0.001	0.001	0.001	0.001	0.001	0.001	0.001	0.004	0.010
Root and tuber vegetable	0.001	0.008	0.001	0.033	0.001	0.012	0.012	0.001	0.001	0.026	0.096
Cucurbitaceae	0.001	0.003	0.001	0.002	0.001	0.002	0.001	0.001	0.001	0.015	0.026
Aquatic vegetable	0.001	0.003	0.000	0.002	0.001	0.001	0.000	0.001	0.000	0.025	0.033
Other vegetables	0.000	0.001	0.000	0.000	0.000	0.000	0.001	0.000	0.000	0.003	0.007
Fruits	0.001	0.001	0.001	0.000	0.001	0.001	0.001	0.000	0.001	0.002	0.007
Citrus	0.001	0.001	0.001	0.001	0.001	0.001	0.001	0.001	0.001	0.004	0.010
Pome	0.001	0.001	0.001	0.001	0.001	0.001	0.001	0.001	0.001	0.002	0.007
Stone fruit	0.001	0.001	0.001	0.000	0.000	0.001	0.000	0.000	0.000	0.002	0.006
Berry fruit	0.000	0.001	0.001	0.001	0.000	0.000	0.000	0.000	0.000	0.003	0.007
Melon fruit	0.000	0.001	0.001	0.000	0.001	0.000	0.001	0.000	0.000	0.002	0.006
Other fruits	0.001	0.001	0.001	0.000	0.001	0.001	0.001	0.000	0.001	0.002	0.007
Meat	0.001	0.001	0.001	0.001	0.000	0.001	0.000	0.001	0.000	0.005	0.010

Food categories	Concentrations (mg/kg)										
	Eu	Gd	Tb	Dy	Ho	Er	Tm	Yb	Lu	Y	Total
Pork and pork products	0.001	0.001	0.001	0.001	0.000	0.001	0.000	0.002	0.000	0.005	0.011
Beef and beef products	0.000	0.001	0.002	0.000	0.001	0.001	0.000	0.001	0.000	0.003	0.007
Mutton and mutton products	0.001	0.001	0.000	0.000	0.000	0.001	0.000	0.002	0.000	0.002	0.007
Chicken and chicken products	0.001	0.001	0.000	0.001	0.000	0.001	0.000	0.000	0.000	0.005	0.008
Other meat and products	0.000	0.000	0.000	0.000	0.000	0.000	0.000	0.000	0.000	0.001	0.001
Liver	0.000	0.003	0.000	0.001	0.000	0.000	0.000	0.000	0.000	0.006	0.011
Kidney	0.000	0.001	0.000	0.001	0.000	0.001	0.000	0.001	0.000	0.009	0.013
Aquatic products	0.003	0.005	0.001	0.004	0.001	0.007	0.001	0.002	0.001	0.047	0.071
Freshwater fishes	0.002	0.003	0.001	0.002	0.001	0.001	0.000	0.001	0.000	0.011	0.021
Marine fishes	0.002	0.004	0.001	0.004	0.001	0.003	0.001	0.004	0.001	0.033	0.054
Freshwater shrimp	0.003	0.004	0.001	0.003	0.001	0.002	0.000	0.001	0.000	0.020	0.034
Marine shrimp	0.005	0.011	0.002	0.007	0.002	0.004	0.001	0.004	0.001	0.445	0.482
Freshwater crab	0.009	0.004	0.001	0.009	0.001	0.002	0.000	0.001	0.001	0.021	0.048
Marine crab	0.005	0.014	0.002	0.007	0.002	0.005	0.001	0.004	0.001	0.052	0.094
Bivalve	0.005	0.024	0.003	0.016	0.003	0.008	0.001	0.010	0.003	0.087	0.160
Cephalopoda	0.001	0.005	0.001	0.004	0.001	0.161	0.000	0.003	0.000	0.069	0.245
Other mollusc	0.002	0.009	0.001	0.006	0.001	0.003	0.000	0.002	0.001	0.037	0.063
Roe viscera	0.001	0.002	0.002	0.001	0.000	0.001	0.000	0.001	0.000	0.007	0.014
Milk	0.001	0.001	0.000	0.001	0.000	0.001	0.000	0.000	0.000	0.006	0.010
Milk powder	0.002	0.003	0.001	0.002	0.001	0.001	0.000	0.001	0.000	0.016	0.027
Liquid milk	0.000	0.000	0.000	0.000	0.000	0.000	0.000	0.000	0.000	0.001	0.002
Eggs	0.002	0.006	0.001	0.005	0.001	0.003	0.000	0.002	0.001	0.039	0.060
Fresh egg	0.001	0.002	0.001	0.002	0.000	0.001	0.000	0.001	0.000	0.015	0.023
Processed egg	0.003	0.014	0.003	0.012	0.002	0.006	0.001	0.005	0.002	0.095	0.142
Thallus	0.006	0.042	0.005	0.151	0.005	0.053	0.043	0.011	0.002	0.205	0.523
Agaric	0.005	0.121	0.003	0.515	0.004	0.165	0.155	0.011	0.002	0.144	1.126
Other fungus	0.006	0.030	0.003	0.150	0.004	0.054	0.045	0.011	0.002	0.165	0.470

Food categories	Concentrations (mg/kg)										
	Eu	Gd	Tb	Dy	Ho	Er	Tm	Yb	Lu	Y	Total
Nori	0.009	0.049	0.014	0.040	0.008	0.020	0.003	0.016	0.002	0.364	0.524
Kelp	0.005	0.031	0.005	0.026	0.005	0.013	0.002	0.010	0.001	0.252	0.349
Other algae	0.009	0.032	0.004	0.021	0.004	0.010	0.001	0.009	0.001	0.120	0.210
Beans	0.009	0.001	0.001	0.001	0.000	0.001	0.000	0.001	0.000	0.006	0.020
Soybean	0.002	0.001	0.000	0.001	0.000	0.001	0.000	0.001	0.000	0.005	0.011
Mung bean	0.006	0.002	0.000	0.001	0.000	0.001	0.000	0.001	0.000	0.005	0.017
Azuki bean	0.021	0.002	0.002	0.001	0.000	0.001	0.000	0.002	0.000	0.009	0.036
Nuts	0.009	0.002	0.002	0.001	0.000	0.001	0.000	0.002	0.000	0.009	0.024
Tea	0.015	0.039	0.007	0.041	0.008	0.025	0.007	0.021	0.004	0.204	0.370
Total	0.003	0.008	0.002	0.010	0.002	0.006	0.002	0.004	0.001	0.045	0.083

Table S7. The mean and P95 exposure of the 13 REEs (except Ce, La, and Y) in the general population and different sex-age groups of the Chinese population (μg/kg BW).

Groups	N	Sc		Pr		Nd		Sm		Eu	
		Mean	P95	Mean	P95	Mean	P95	Mean	P95	Mean	P95
2-6 years	4239	0.37	1.55	0.07	0.18	0.27	0.70	0.05	0.14	0.02	0.09
7-12 years	3256	0.25	0.58	0.06	0.17	0.23	0.66	0.04	0.13	0.02	0.07
13-17 years males	888	0.20	0.41	0.05	0.11	0.18	0.41	0.03	0.08	0.012	0.05
13-17 years females	824	0.19	0.47	0.04	0.11	0.18	0.42	0.03	0.08	0.01	0.04
≥18 years males	24210	0.17	0.48	0.04	0.12	0.17	0.45	0.03	0.10	0.01	0.04
≥18 years females	28394	0.17	0.48	0.04	0.13	0.17	0.51	0.03	0.10	0.01	0.04
Total	61811	0.17	0.56	0.04	0.11	0.16	0.41	0.03	0.10	0.01	0.04

Groups	N	Gd		Tb		Dy		Ho		Er	
		Mean	P95	Mean	P95	Mean	P95	Mean	P95	Mean	P95
2-6 years	4239	0.062	0.31	0.01	0.06	0.12	1.23	0.01	0.04	0.05	0.77
7-12 years	3256	0.051	0.25	0.01	0.03	0.09	0.99	0.02	0.04	0.04	0.38
13-17 years males	888	0.042	0.23	0.01	0.02	0.08	0.90	0.01	0.03	0.04	0.35
13-17 years females	824	0.040	0.24	0.01	0.02	0.08	0.97	0.01	0.02	0.03	0.53
≥18 years males	24210	0.038	0.17	0.01	0.02	0.07	0.67	0.01	0.02	0.03	0.36
≥18 years females	28394	0.038	0.18	0.01	0.02	0.07	0.72	0.01	0.03	0.03	0.31
Total	61811	0.036	0.18	0.01	0.02	0.07	0.72	0.01	0.02	0.03	0.34

Groups	N	Tm		Yb		Lu	
		Mean	P95	Mean	P95	Mean	P95
2-6 years	4239	0.03	0.37	0.02	0.13	0.01	0.02
7-12 years	3256	0.03	0.30	0.02	0.14	0.01	0.01
13-17 years males	888	0.02	0.27	0.02	0.09	0.01	0.01
13-17 years females	824	0.02	0.29	0.02	0.08	0.01	0.01
≥18 years males	24210	0.02	0.20	0.01	0.08	0.00	0.01
≥18 years females	28394	0.02	0.22	0.01	0.08	0.00	0.01
Total	61811	0.02	0.23	0.01	0.08	0.00	0.01
